# Comparative Efficacy of Neurofeedback Interventions for Attention‐Deficit/Hyperactivity Disorder in Children: A Network Meta‐Analysis

**DOI:** 10.1002/brb3.70194

**Published:** 2024-12-22

**Authors:** Gang Wu, Qiang He, Da Li, Zhang Zhang, Jinli Miao, Yanping Shu

**Affiliations:** ^1^ Department of Psychiatry of Women and Children The Second People's Hospital of Guizhou Province Guiyang Guizhou China; ^2^ The Yangtze River Delta Biological Medicine Research and Development Center of Zhejiang Province Yangtze Delta Region Institution of Tsinghua University Hangzhou Zhejiang China

**Keywords:** ADHD, children, neurofeedback interventions, nonpharmacological strategies

## Abstract

**Objective:**

This study aimed to synthesize and encapsulate findings from recent research (May 1, 2018 to August 1, 2023) on neurofeedback interventions for children diagnosed with attention deficit hyperactivity disorder (ADHD).

**Methods:**

A comprehensive search was conducted across major databases and platforms, including randomized controlled trials s focusing on children aged 5–11 years with ADHD. The inclusion was broad, not restricted by ADHD subtype, gender, IQ, socioeconomic status, or coexisting conditions.

**Results:**

From the study screening process, 13 studies were included in the network meta‐analysis, involving 1370 children. Most neurofeedback therapies surpassed placebo in ADHD symptoms. In the acceptability outcome, five neurofeedback therapies (HEG, SCP training, TBR training, SMR training, and active control) outperformed the inactive control, physical activity, and EMG therapies.

**Conclusions:**

The potential efficacy of nonpharmacological interventions in ADHD management among children is illuminated. The findings advocate for a holistic, child‐centered approach, emphasizing the need for further in‐depth research to understand and refine these interventions.

## Graphical abstract

This network meta‐analysis revealed that nonpharmacological interventions, especially neurofeedback therapies (HEG, SCP, TBR, SMR, and active control) can significantly improve ADHD symptoms in children, advocating a holistic child‐centered management approach.

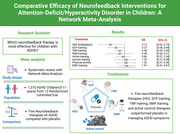

## 1 Introduction

Attention deficit hyperactivity disorder (ADHD) is a prevalent neurodevelopmental disorder characterized by symptoms of impulsivity, hyperactivity, and inattention, primarily affecting children (Thapar and Cooper 2016). Epidemiological data illustrate that the incidence of ADHD is threefold higher in boys compared to girls (Fombonne 1994). ADHD not only impacts academic performance and development but also often persists into adulthood, associated with educational underachievement, interpersonal difficulties, and mental health issues (Franke, Michelini, et al. 2018). ADHD's etiology is complex, involving genetic, psychological, and environmental factors (Demontis et al. 2023; Thapar and Cooper 2016; Sciberras et al. 2017). Empirical evidence indicates a deficiency in executive functioning, impacting attention regulation, planning, and working memory (Thapar and Cooper 2016). Currently, the main therapies for ADHD include pharmacological and behavioral therapies (Caye et al. 2019). While psychostimulant medications such as Ritalin and modafinil are commonly prescribed, they are associated with side effects such as insomnia and mood swings (Pakdaman et al. 2018). In addition to pharmacological treatments, the incorporation of nonpharmacological modalities such as behavioral therapy, psychoeducation, and social skills training anchored in neurofeedback is essential for a comprehensive enhancement of children's functional and qualitative life outcomes (Austerman 2015). Nonetheless, clinical evidence for these interventions is not yet exhaustive, underscoring the necessity for ongoing research to elucidate their efficacy and optimize ADHD management protocols.

To seek for therapies with fewer side effects, an increased focus has been directed toward nonpharmacological interventions including neurofeedback and cognitive behavioral therapies (CBTs; Enriquez‐Geppert et al. 2019; Sciberras et al. 2019). While these strategies have demonstrated potential in ameliorating cognitive and behavioral impairments associated with ADHD, empirical evidence substantiating their definitive impact remains inconclusive. Customized educational and behavioral interventions deployed within school and summer program settings have demonstrated efficacy in facilitating the academic and social adaptation of students with ADHD (DuPaul et al. 2014). Moreover, neurofeedback, which entails the modulation of brain wave activity, has been implicated in enhancing attention and impulse control, although the extent of its efficacy is subject to ongoing investigation. For example, the efficacy of the widely debated theta–beta power ratio (TBR) neurofeedback training was confirmed in a recent clinical study (Neurofeedback Collaborative 2023), along with other approaches like slow cortical potential (SCP) and sensorimotor rhythm (SMR) neurofeedback (Heinrich et al. 2019; Krepel et al. 2020). CBT aids ADHD individuals in recognizing and modifying maladaptive patterns, thereby enhancing their functional abilities (Pan et al. 2019; Mueller et al. 2017). Notwithstanding the promising prospects of these nonpharmacological interventions, a comprehensive body of research delineating their efficacy, safety, and optimal implementation protocols is necessitated. This will enable the formulation of robust, evidence‐based recommendations for the holistic management of ADHD, bridging the extant gaps in our understanding and offering diversified, effective therapeutic options.

Numerous systematic reviews and meta‐analyses have rigorously examined the impacts of nonpharmacological interventions on ADHD (Cortese et al. 2018; Samea et al. 2019; Sun et al. 2022). Despite the prevalent application of these treatment modalities, a paucity of directly comparable clinical trials engenders ambiguity regarding their efficacy and safety. This ambiguity is exacerbated by limited emergent evidence within the preceding 5 years. Network meta‐analysis combines both direct and indirect evidence to evaluate the efficacy and safety of various treatments. It helps clinicians and policies make evidence‐based decisions. This tool is crucial for assessing diverse treatment options, especially in therapies like neurofeedback for pediatric ADHD patients (Li et al. 2023). This study aims to conduct a network meta‐analysis to compare the efficacy of different neurofeedback therapies for children with ADHD, emphasizing the importance of a collaborative approach in treatment optimization.

## 2 Methods

### 2.1 Search Strategy

This systematic review was conducted in compliance with the guidelines provided Reporting Items for Systematic Reviews and Meta‐Analyses (PRISMA) statement (Hutton et al. 2015). Comprehensive searches were executed across prominent databases and platforms including PubMed, Cochrane Central Register of Controlled Trials (CCRCT), EMBASE, MEDLINE, and the WHO International Trials Registry Platform, which encompasses ClinicalTrials.gov. These relevant studies were published from May 1, 2018 to August 1, 2023. The impetus for our review emanated from two antecedent studies of a similar nature (Catala‐Lopez et al. 2015, 2017). We limit the language to English, aiming for an inclusive and exhaustive representation of the global research landscape. Our inquiry utilized specific terms, including (“attention deficit disorder with hyperactivity”) AND (“neurofeedback” OR “biofeedback” OR “neurofeedback” OR “EEG biofeedback” OR “brainwave training”) AND (“child”). In addition to the systematic interrogation of electronic databases, we engaged in direct correspondence with study authors. Data extraction was conducted by a minimum of two independent researchers.

### 2.2 Inclusion and Exclusion Criteria

Inclusion criteria include the following: (1) studies based on double‐blind randomized controlled trials (RCTs) with a duration of at least 1 week, a minimum follow‐up period of 3 weeks, (2) children aged 5–11 years were diagnosed with ADHD according to DSM‐III, DSM III‐R, DSM‐IV(TR), DSM‐5, ICD‐9, or ICD‐10 criteria, (3) studies that included evaluating various neurofeedback therapies, (4) all ADHD subtypes, and (5) studies that involved patients with comorbidities.

Exclusion criteria include the following: (1) studies that incorporated pharmaceutical interventions, (2) the follow‐up period was less than 3 weeks, (3) studies with unavailable full text and conference abstract, (4) studies with insufficient data, and (5) studies that were irrelevant to the topic.

### 2.3 Risk of Bias Assessment

The assessment of the risk of bias was systematically executed employing the Cochrane risk‐of‐bias tool for randomized trials (Risk of Bias 2, http://methods.cochrane.org/bias/). In addition, the certainty of the amassed evidence was appraised using the Grading of Recommendations Assessment, Development, and Evaluation (GRADE) methodology (Higgins et al. 2014).

### 2.4 Statistical Analysis

In the primary analyses, we focused on assessing efficacy, specifically, changes in clinician ratings of the severity of core ADHD symptoms in children. If studies did not incorporate the Attention‐Deficit/Hyperactivity Disorder Rating Scale (ADHD‐RS), we systematically employed the Conners Rating Scale (encompassing any version, ADHD total score), or alternative ADHD scales as suitable replacements. We evaluated subscales that independently assessed the distinct dimensions of inattention and hyperactivity/impulsivity symptoms. Studies not reporting these measures were excluded, ensuring only relevant data contributed to our findings. For secondary outcomes, we considered the acceptability of children's participation by evaluating the proportion of participants who withdrew for various reasons. This assessment was instrumental in gauging the overall feasibility and tolerability of the interventions, offering invaluable insights into the practical applicability of the treatment modalities under scrutiny.

We conducted meta‐analyses of all outcomes and comparisons, examining multiple neurofeedback therapies versus placebo, utilizing a random effects model. We computed standardized mean differences, Cohen's *d*, odds ratios, and their corresponding 95% confidence intervals (CIs) for both continuous and dichotomous outcomes. The *I* ^2^ value and its 95% CI, *τ* ^2^, and *Q* value were calculated to assess statistical and between‐study heterogeneity. Subsequently, a network meta‐analysis was executed within a frequentist framework. Our assessment extended to both direct and indirect evidence. Sensitivity analysis was also undertaken, encompassing multiple meta‐regression to evaluate the impact of clinical and study design effect modifiers. These included the year of publication, sex, age, and number of centers participating in the study. This thorough analysis enabled a comprehensive assessment of various factors' influence on the model. All analyses were performed utilizing R software, version 4.3.1.

## 3 Results

**FIGURE 1:**
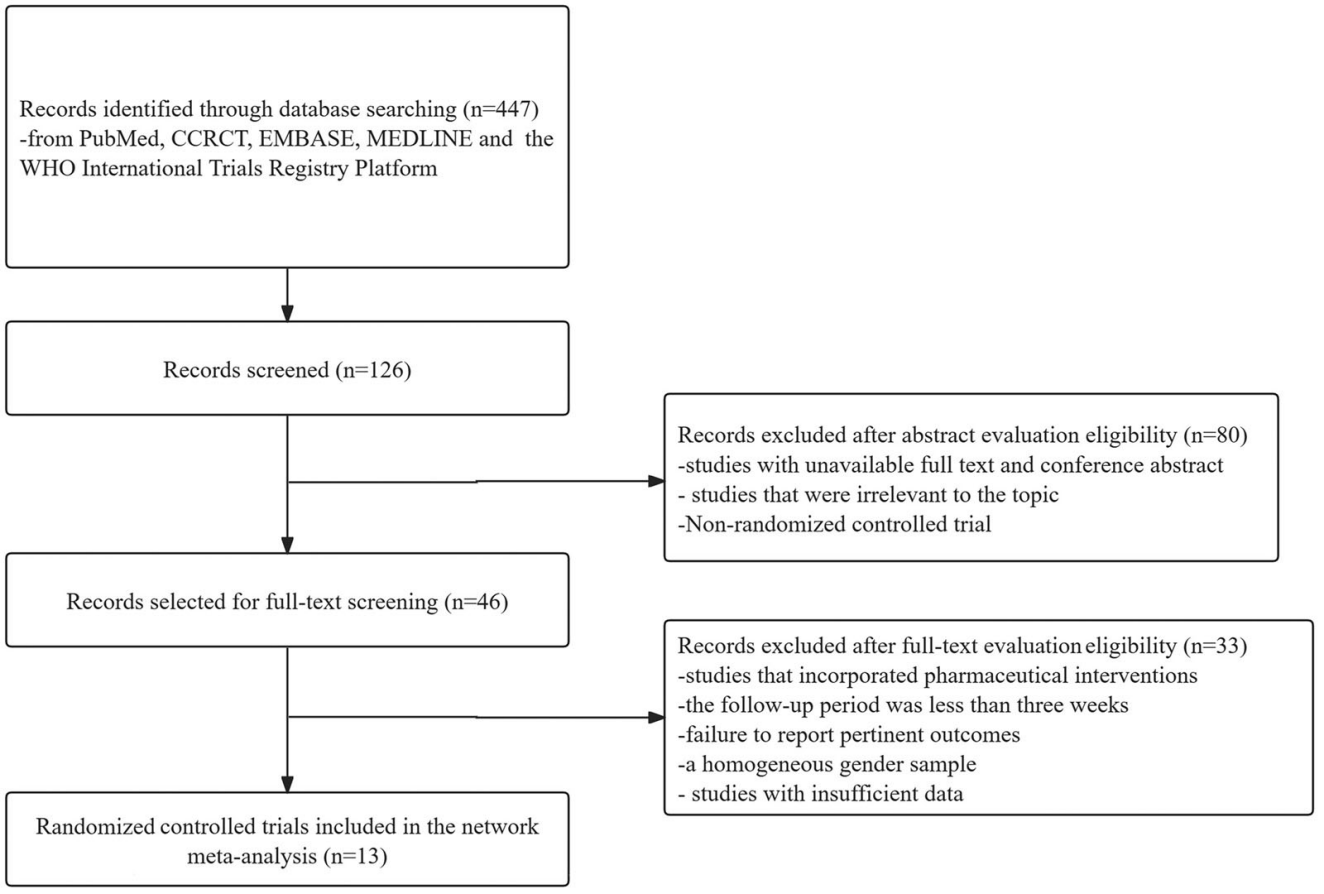
Flowchart of literature screening.

The literature search, study selection, and data extraction processes were executed from January 1, 2023 to June 1, 2023. Data analysis was subsequently completed between June 2, 2023 and September 30, 2023. Figure 1 illustrates the study screening process, from which 13 studies (Aggensteiner et al. 2019; Baumeister et al. 2019; Gevensleben et al. 2020; Ghadamgahi Sani et al. 2022; Korfmacher et al. 2022; Liao et al. 2022; Luo et al. 2023; Neurofeedback Collaborative 2021, 2023; Ning and Wang 2021; Purper‐Ouakil et al. 2022; Rahmani et al. 2022; Skalski 2022) were ultimately retained for the network meta‐analysis, involving a total of 1370 children. A significant 89.7% of the initial studies were excluded due to reasons such as the nature of the study, failure to report pertinent outcomes, and a homogeneous gender sample.

**FIGURE 2:**
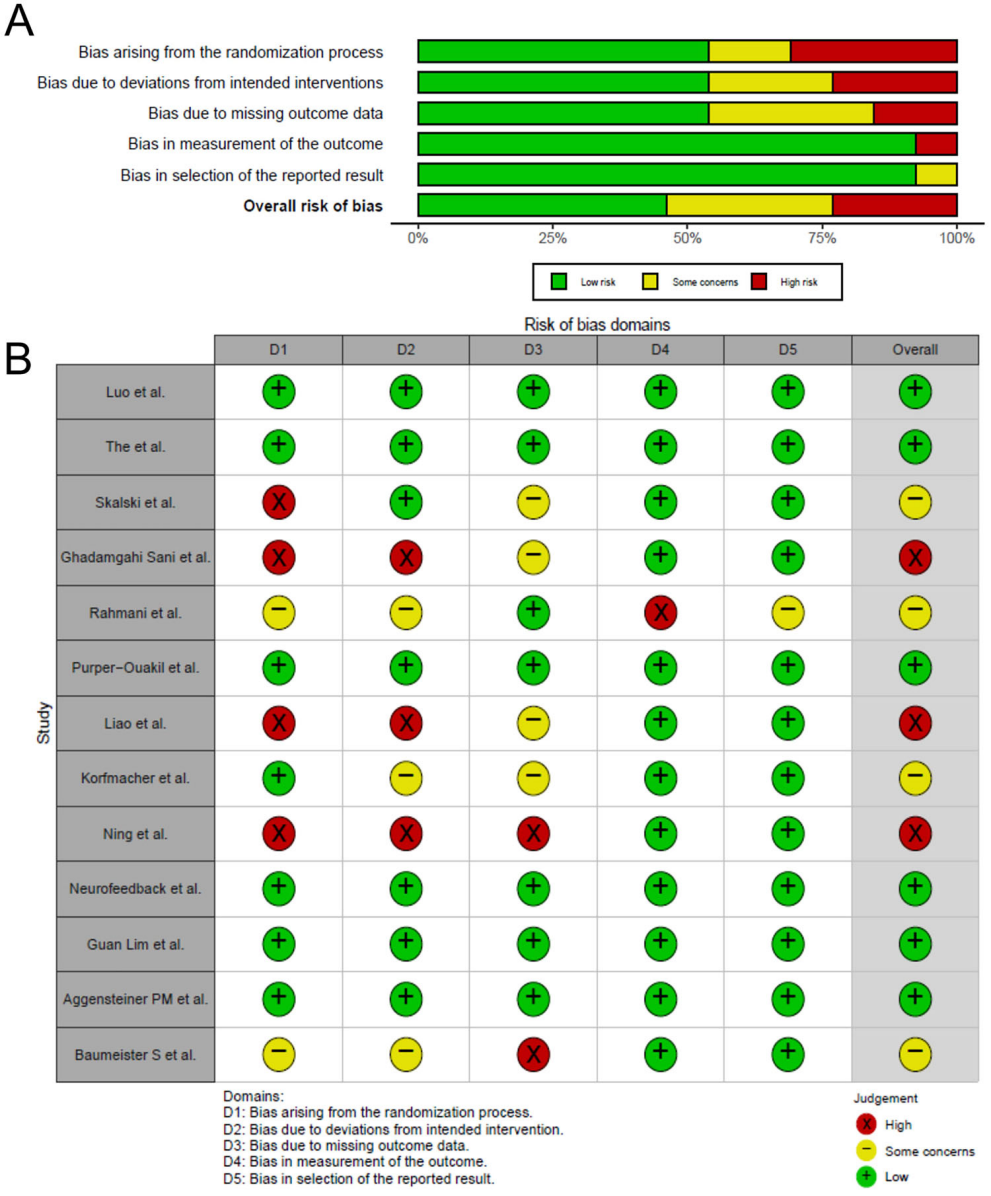
Results of risk assessment of included studies. (A) bar chart; (B) traffic chart. Red, yellow, and green represent high, medium, and low levels of risk in that order.

We then assessed the risk of bias for the final 13 included studies. Of the 13 studies included, and all of which were RCTs, a total of 1370 children were enrolled, whose mean age was 9.48 ± 1.28 years, with 54.5% being boys. The DSM‐IV/V was used for diagnosis, and only one study used the Swanson, Nolan, and Pelham Teacher and Parent Rating Scale IV (SNAP‐IV), a widely used scale for evaluating ADHD symptoms, which is an extension of the DSM evaluation system. Only eight studies (Aggensteiner et al. 2019; Baumeister et al. 2019; Ghadamgahi Sani et al. 2022; Korfmacher et al. 2022; Liao et al. 2022; Luo et al. 2023; Neurofeedback Collaborative 2021; Purper‐Ouakil et al. 2022) reported children's IQ and five studies (Gevensleben et al. 2020; Korfmacher et al. 2022; Neurofeedback Collaborative 2021, 2023; Ning and Wang 2021) reported subtypes of ADHD. Treatment cycles ranged from 20 h to 25 months. In evaluating the risk of bias, 46.2% of the child‐centered studies exhibited a low risk, 30.8% an unclear risk, and 23.1% a high risk of bias (Figure 2A,B).

**FIGURE 3:**
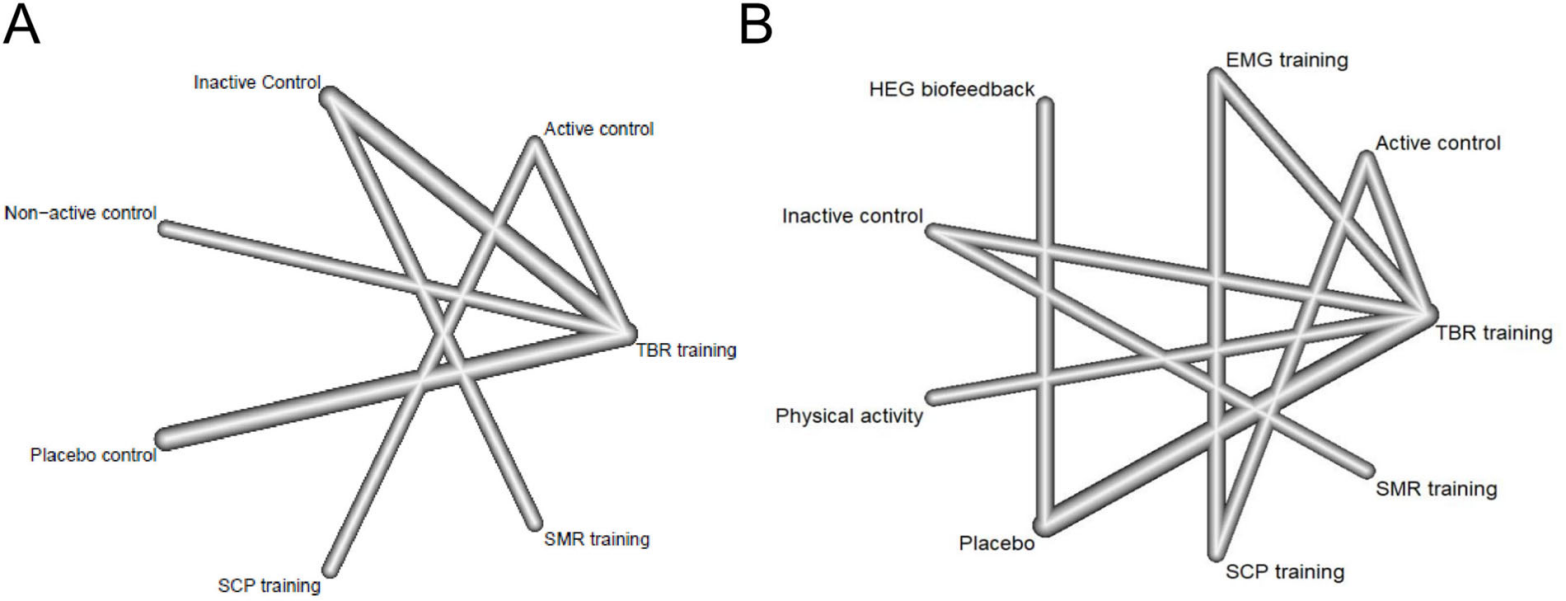
Network structure graph for ADHD‐RS (A) and acceptability (B). The width of the line is proportional to the number of trials comparing each treatment pair, and the size of each circle is proportional to the number of participants randomly assigned (sample size). Placebo control (sham neurofeedback).

We conducted a network meta‐analysis comparing different ADHD interventions. For the ADHD‐RS, we analyzed seven therapies (six of them based on neurofeedback and one on placebo). The connecting lines represent direct comparisons of the two therapies, and it can be seen that the thicker lines between inactive control and TBR training and placebo and TBR training indicate a higher frequency of comparisons between these two groups (Figure 3A). And in the network structure diagram comparing acceptability, there were nine therapies (eight neurofeedback‐based therapies as well as one placebo.) The frequency of comparisons between Placebo and TBR training was higher (Figure 3B). No multiarm trials were compared among these comparisons.

**FIGURE 4:**
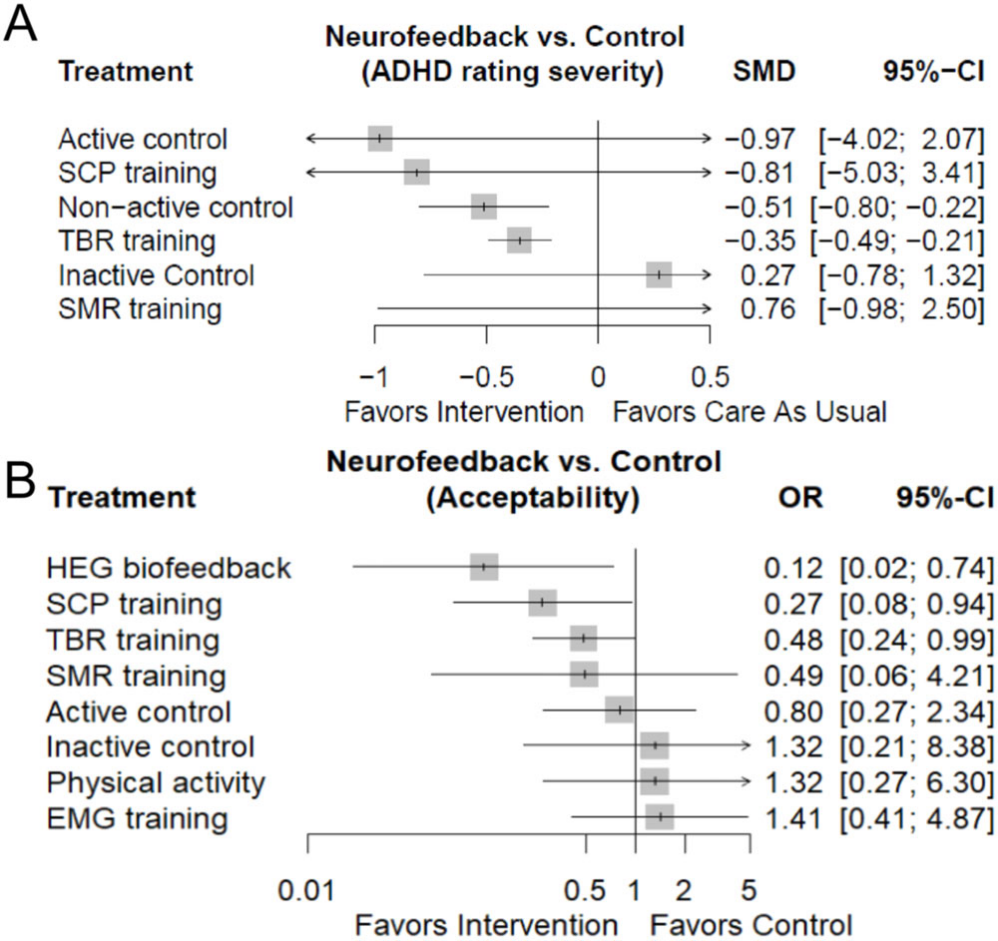
The forest plot includes ADHD‐RS (A) and acceptability (B) and is compared with placebo as a reference. ACT, active control (medication, e.g., methylphenidate) OR self‐management training; ADHD‐RS, Attention Deficit Hyperactivity Disorder rating scale; CI, confidence interval; EMG, electromyographic biofeedback; HEG BFB, hemoencephalographic biofeedback; INC, inactive control (waiting list controls, in which the children do nothing); NAC, nonactive control (waiting list controls, attention training, cognitive training, treatment as usual, sham control); OR, odds ratio; PA, physical activity; SCP, slow cortical potential training; SMD, standardized mean difference; SMR, sensorimotor rhythm neurofeedback training; TBR, theta/beta neurofeedback training.

**FIGURE 5:**
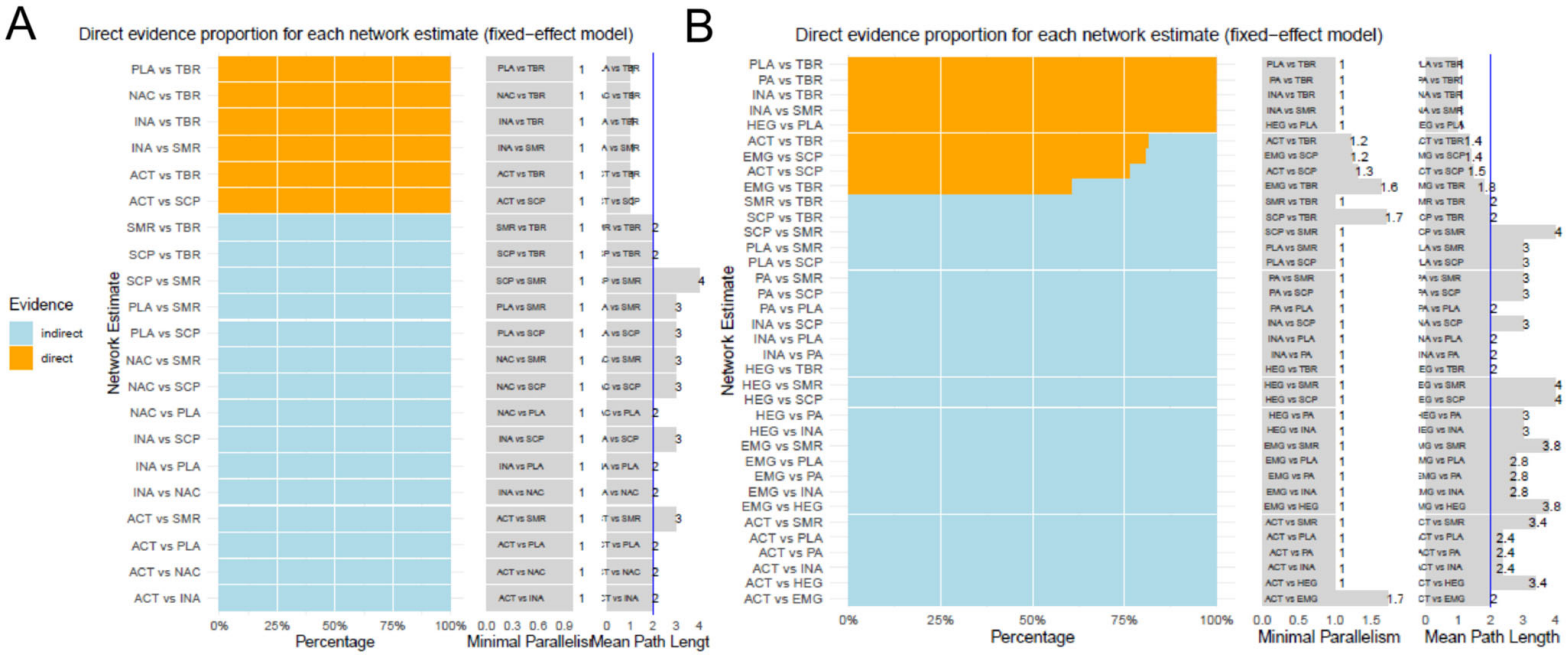
Comparison across studies on ADHD‐RS (A) and acceptability (B) direct and indirect evidence profiles.

We then performed a network meta‐analysis of the effects of different neurofeedback therapies. For the ADHD‐RS, almost all neurofeedback therapies outperformed placebo, except for inactive control, and in the acceptability outcome, five neurofeedback therapies (HEG, SCP training, TBR training, SMR training, and active control) outperformed inactive control, physical activity, and EMG (Figure 4A). In the acceptability outcome, five neurofeedback therapies (HEG, SCP training, TBR training, SMR training, and active control) outperformed inactive control, physical activity, and EMG therapies (Figure 4B). While Tables 1 and 2 present the effect size estimates for each possible treatment comparison, Table 1 demonstrates the results in terms of “ADHD‐RS” and therapies such as TBR training, SCP training, and so forth all performed better relative to placebo, inactive control, and so forth. Table 2 demonstrates the results in terms of acceptability, and the results are similar to the situation in Table 1, and it is worth noting that HEG has a much higher effect relative to EMG. However, Figure 5A shows that in terms of ADHD‐RS, most comparisons were indirect, whereas direct comparisons were more frequent in the included studies. Figure 5B shows that in terms of acceptability, there were still a large number of nonindirect comparisons. These results suggest a requisite for a more substantial body of evidence from indirect comparisons.

**TABLE 1:** Effect table for ADHD‐RS.

	ACT	INA	NAC	PLA	SCP	SMR	TBR
ACT	—	—	—	—	−0.16 (−3.09; 2.76)	—	−0.62 (−3.66; 2.42)
INA	−1.25 (−4.46; 1.97)	—	—	—	—	−0.49 (−1.87; 0.90)	0.62 (−0.42; 1.66)
NAC	−0.46 (−3.51; 2.59)	0.78 (−0.29; 1.85)	—	—	—	—	−0.16 (−0.41; 0.10)
PLA	−0.97 (−4.02; 2.07)	0.27 (−0.78; 1.32)	−0.51 (−0.80; −0.22)	—	—	—	0.35 (0.21; 0.49)
SCP	−0.16 (−3.09; 2.76)	1.08 (−3.26; 5.43)	0.30 (−3.92; 4.53)	0.81 (−3.41; 5.03)	—	—	—
SMR	−1.73 (−5.23; 1.77)	−0.49 (−1.87; 0.90)	−1.27 (−3.02; 0.48)	−0.76 (−2.50; 0.98)	−1.57 (−6.13; 2.99)	—	—
TBR	−0.62 (−3.66; 2.42)	0.62 (−0.42; 1.66)	−0.16 (−0.41; 0.10)	0.35 (0.21; 0.49)	−0.46 (−4.68; 3.76)	1.11 (−0.62; 2.85)	—

**TABLE 2:** Effect table for acceptability.

	ACT	EMG	HEG	INA	PA	PLA	SCP	SMR	TBR
ACT	—	—	—	—	—	—	2.66 (0.98; 7.24)	—	1.79 (0.74; 4.31)
EMG	0.57 (0.20; 1.57)	—	—	—	—	—	5.64 (2.29; 13.90)	—	2.46 (0.67; 8.97)
HEG	6.73 (0.81; 55.81)	11.86 (1.31; 107.48)	—	—	—	0.12 (0.02; 0.74)	—	—	—
INA	0.61 (0.09; 3.99)	1.07 (0.15; 7.77)	0.09 (0.01; 1.21)	—	—	—	—	2.69 (0.90; 8.07)	2.72 (0.49; 14.96)
PA	0.61 (0.12; 3.02)	1.07 (0.19; 5.97)	0.09 (0.01; 1.00)	1.00 (0.11; 9.03)	—	—	—	—	2.72 (0.68; 10.93)
PLA	0.80 (0.27; 2.34)	1.41 (0.41; 4.87)	0.12 (0.02; 0.74)	1.32 (0.21; 8.38)	1.32 (0.27; 6.30)	—	—	—	2.07 (1.01; 4.24)
SCP	2.95 (1.23; 7.07)	5.20 (2.31; 11.69)	0.44 (0.05; 3.99)	4.85 (0.67; 35.35)	4.85 (0.87; 27.21)	3.69 (1.06; 12.82)	—	—	—
SMR	1.63 (0.18; 14.45)	2.88 (0.30; 27.76)	0.24 (0.01; 4.08)	2.69 (0.90; 8.07)	2.69 (0.23; 31.48)	2.04 (0.24; 17.59)	0.55 (0.06; 5.36)	—	—
TBR	1.65 (0.74; 3.67)	2.91 (1.06; 7.98)	0.25 (0.03; 1.74)	2.72 (0.49; 14.96)	2.72 (0.68; 10.93)	2.07 (1.01; 4.24)	0.56 (0.20; 1.55)	1.01 (0.13; 7.67)	—

*Note*: Data are shown with mean, 95% CI.

Abbreviations: ACT, active control (medication, e.g., methylphenidate) OR self‐management training; EMG, electromyographic biofeedback; HEG BFB, hemoencephalographic biofeedback; INC, inactive control (waiting list controls, in which the children do nothing); NAC, nonactive control (waiting list controls, attention training, cognitive training, treatment as usual, sham control); PA, physical activity; SCP, slow cortical potential training; SMR, sensorimotor rhythm neurofeedback training; TBR, theta/beta neurofeedback training.

Sensitivity analyses incorporating multiple meta‐regression analyses were conducted to scrutinize the impacts of variables including the gender, the number of centers, year of publication, age, treatment duration involved in the studies, assessing potential multicollinearity (Table 3; Figures S1 and S2). The model shows that all predictor variables do not have significant multicollinearity and do not have a significant effect on the prediction. It is worth noting that age is potentially the best predictor variable, followed by gender, the number of centers, year of publication, and treatment duration. The analyses' outcomes were generally robust. However, limitations arose due to the paucity of data, rendering us incapable of evaluating the effects of factors like IQ, treatment period, risk of bias, and medication status comprehensively.

**TABLE 3:** Multiple meta‐regression analysis to analyze robustness of predictors.

	Estimate	SE	*t*val	df	*p*val	ci.lb	ci.ub
Intercept	1.1632	3.7252	0.3122	2	0.7844	14.8652	17.1915
Gender	−0.0178	0.0154	−1.1492	2	0.3694	−0.0842	0.0487
Centers	0.011	0.1637	0.0671	2	0.9526	−0.6934	0.7154
Year	−0.336	0.1616	−2.0789	2	0.1732	−1.0314	0.3594
Age	0.0014	0.2877	0.0048	2	0.9966	−1.2366	1.2393
Duration	0.0452	0.0202	2.2423	2	0.1542	−0.0416	0.132

Abbreviations: ci.lb, 95% confidence interval lower bound; ci.ub, 95% confidence interval upper bound; df, degrees of freedom; *P*val, *p* value; SE, standard error;. Tval, *t* value.

## 4 Discussion

Our network meta‐analysis embarks on an in‐depth examination of nonpharmacological interventions for ADHD among children, addressing a significant gap in existing literature that predominantly focuses on medication‐based approaches. The meticulous endeavor carried out from January 1, 2023 to September 30, 2023, facilitated a discerning selection of 13 studies encompassing 1370 children, thereby providing a robust platform for evaluating the comparative efficacy of these interventions. Unlike previous studies, which have shown inconsistent effects of nonpharmacological interventions on ADHD symptoms (Sibley et al. 2023), our meta‐analysis explores variety of interventions, unveiling a more nuanced understanding of their potential benefits in managing ADHD symptoms among children. This work not only broadens the scope of existing knowledge but also supports a more holistic approach to managing ADHD beyond conventional medication centric.

In evaluating the risk of bias, we found that 46.2% of the studies manifested a low risk, 30.8% an unclear risk, while 23.1% exhibited a high risk of bias. This analysis echoes the sentiments in contemporary research that underline the necessity for rigorous methodologies to mitigate sources of bias and enhance the robustness of findings (Goode et al. 2018; Ogundele and Ayyash 2023). Our risk assessment not only indicates the potential avenues for enhancing the quality of future investigations but also augments the integrity and credibility of findings in this domain. Comparatively, our methodological rigor in selecting studies and assessing the risk of bias underscores a significant advancement in ensuring the reliability and validity of the outcomes, thus, contributing to the burgeoning literature on nonpharmacological interventions for ADHD in children (Qiu et al. 2023).

Our investigation provides further evidence for the nonpharmacological interventions for ADHD in children. A salient revelation is the pronounced efficacy of most interventions versus placebo controls in ameliorating clinician‐rated core ADHD symptoms. This aligns with the broader discourse advocating for a multimodal strategy in managing ADHD, where nonpharmacological interventions play a crucial role alongside medication (Mechler et al. 2022). The data show a significant increase in acceptance for 10 interventions (active control [self‐managing training], nonactive control [cognitive training], inactive control [waiting list], TBR training, SCP training, SMR training, physical activity, EMG therapies, and HEG BFB), underscoring their promising prospects in clinical settings. Unlike medication, which offers immediate but transient effects, nonpharmacological interventions may offer a more sustainable approach to managing ADHD symptoms (Ogundele and Ayyash 2023). Our findings are in concert with existing literature that also underscores the potential of structured behavioral interventions and complementary strategies in managing ADHD (Goode et al. 2018; Shrestha, Lautenschleger, and Soares 2020).

Comparative analysis with existing literature provides a nuanced understanding of the potential of these interventions in addressing both the core symptoms and the behavioral and other related difficulties that children with ADHD often encounter. The analytical framework employed in our study provides a robust basis for future explorations aimed at distilling optimal nonpharmacological strategies for managing ADHD in children. This comparison reinforces the importance of integrating nonpharmacological interventions in ADHD management and highlights the need for more detailed research to determine the most effective approaches. The delineation of intervention components and features associated with effective change, as illustrated in some studies (Russell et al. 2022), also accentuates the importance of a tailored approach, thus, enriching the discourse on nonpharmacological interventions for ADHD among children.

The sensitivity analysis including multiple meta‐regressions allowed for a thorough examination of variables such as publication year, gender, age, the duration of treatment, and the number of centers partaking in the studies. Although the analyses' outcomes were robust, showing no significant instances of covariance, a notable limitation was the scant data on certain critical variables such as IQ, treatment duration, risk of bias, and medication status. This data scarcity aligns with earlier research that also underscored data insufficiency as a limiting factor in comprehensively evaluating the effects of nonpharmacological interventions (Sibley et al. 2023). Our study contributes to the discourse by highlighting the need for more expansive data collection in subsequent studies to foster a more nuanced understanding of the variables affecting the efficacy of nonpharmacological interventions for ADHD in children. Moreover, identifying potential multicollinearity in our analyses signifies an advanced methodological approach, thereby contributing to the broader endeavor of elucidating the complex dynamics underpinning nonpharmacological interventions for ADHD.

In summation, our analysis provides a significant exploration of nonpharmacological interventions for ADHD in children, transcending the conventional medication‐centric paradigm. The findings enhance our understanding of the nonpharmacological interventions' efficacy and advocate for a shift toward more holistic, child‐centric ADHD management strategies (Weibel et al. 2020). This narrative aligns with the growing body of literature advocating for a multimodal approach to ADHD management, which encompasses both pharmacological and nonpharmacological strategies to address the multifaceted nature of ADHD (Goode et al. 2018; Ogundele and Ayyash 2023). Our study illuminates promising avenues while underscoring the exigency for more exhaustive research, especially concerning the identified limitations. The call for refining nonpharmacological interventions and the propulsion of the discourse toward more effective, child‐focused ADHD treatment modalities resonate with the broader research goals. Moreover, synthesizing existing evidence suggests a need for a toolkit of nonpharmacological strategies that school staff can use to improve the primary school experience for children with ADHD (Shrestha, Lautenschleger, and Soares 2020). The insights garnered from this study contribute to the evolving narrative on ADHD management, providing a robust platform for future research aimed at enhancing the well‐being and developmental outcomes of children with ADHD.

## 5 Conclusion

Our study illuminates the potential efficacy of nonpharmacological interventions in managing ADHD among children, advocating for a more holistic, child‐centered approach. The identified limitations underscore the imperative for further comprehensive research, fostering a more nuanced understanding of nonpharmacological interventions and advancing the discourse toward tailored ADHD management strategies.

## Author Contributions

**Gang Wu**: conceptualization, writing–original draft, methodology, software. **Qiang He**: software, methodology. **Da Li**: software, methodology. **Zhang Zhang**: visualization, data curation. **Jinli Miao**: visualization, data curation. **Yanping Shu**: conceptualization, writing–review and editing, writing–original draft.

## Ethics Statement

The authors have nothing to report.

## Conflicts of Interest

The authors declare no conflicts of interest.

### Peer Review

The peer review history for this article is available at https://publons.com/publon/10.1002/brb3.70194

## Supporting information



Supplementary Materials.

## Data Availability

All data generated or analyzed during this study are included in this published article and its supplementary information files.
