# Supplementary material for: Comparative Efficacy of Neurofeedback Interventions for Attention‐Deficit/Hyperactivity Disorder in Children: A Network Meta‐Analysis
Source: Brain Behav. 2024 Dec 22;14(12):e70194. doi: 10.1002/brb3.70194 (PMC11664034; doi:10.1002/brb3.70194)
Supplement: Supplementary file 1 — Supplementary Materials. [file BRB3-14-e70194-s001.docx]

**Supplementary figure 1.** The correlation matrix of multi-collinearity


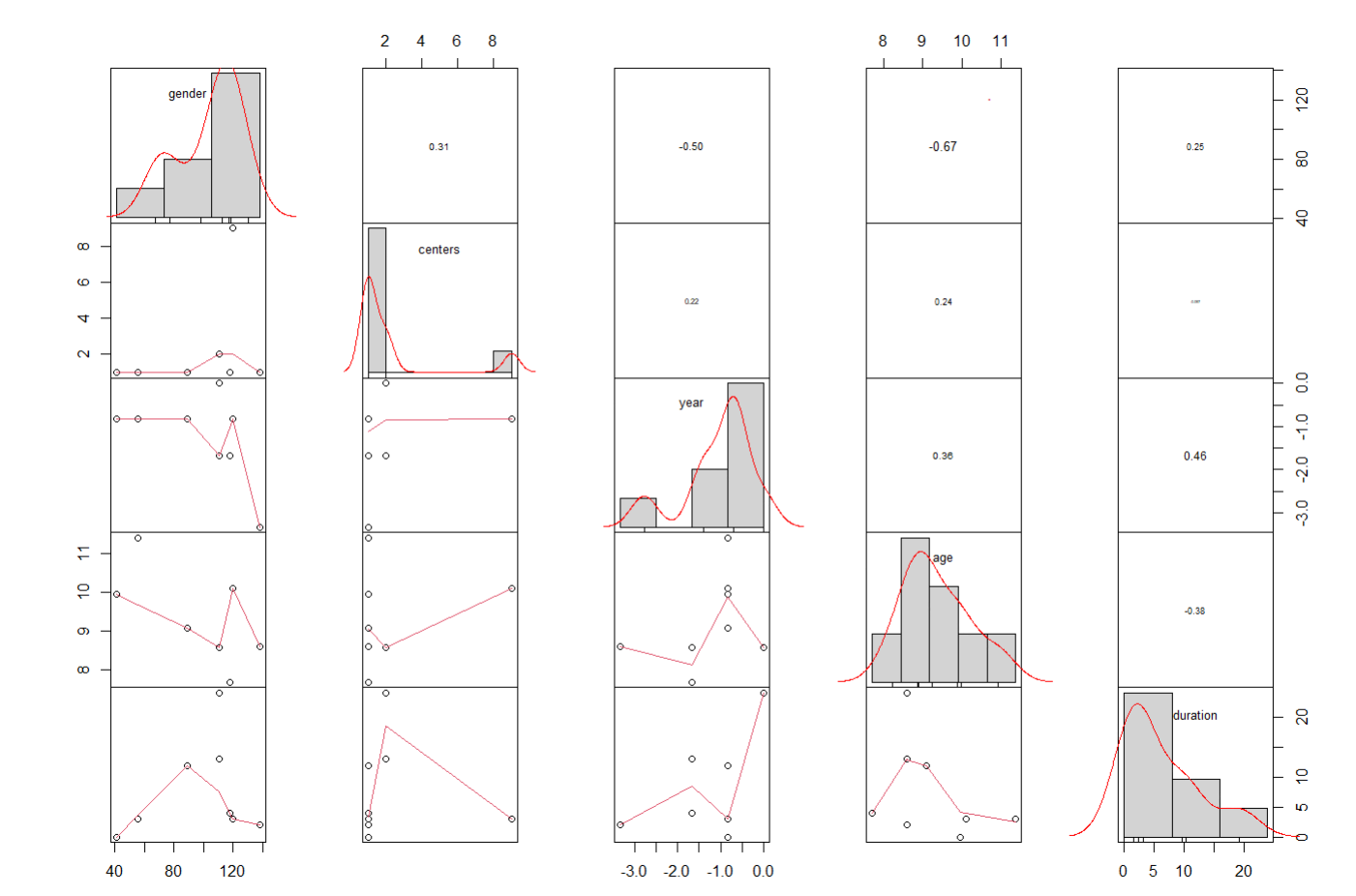


An inter-correlation matrix was constructed after checking for multi-collinearity of the predictors to ensure the robustness of the meta-regression coefficient estimates.

**Supplementary figure 2.** Multi-model inference of best predictors


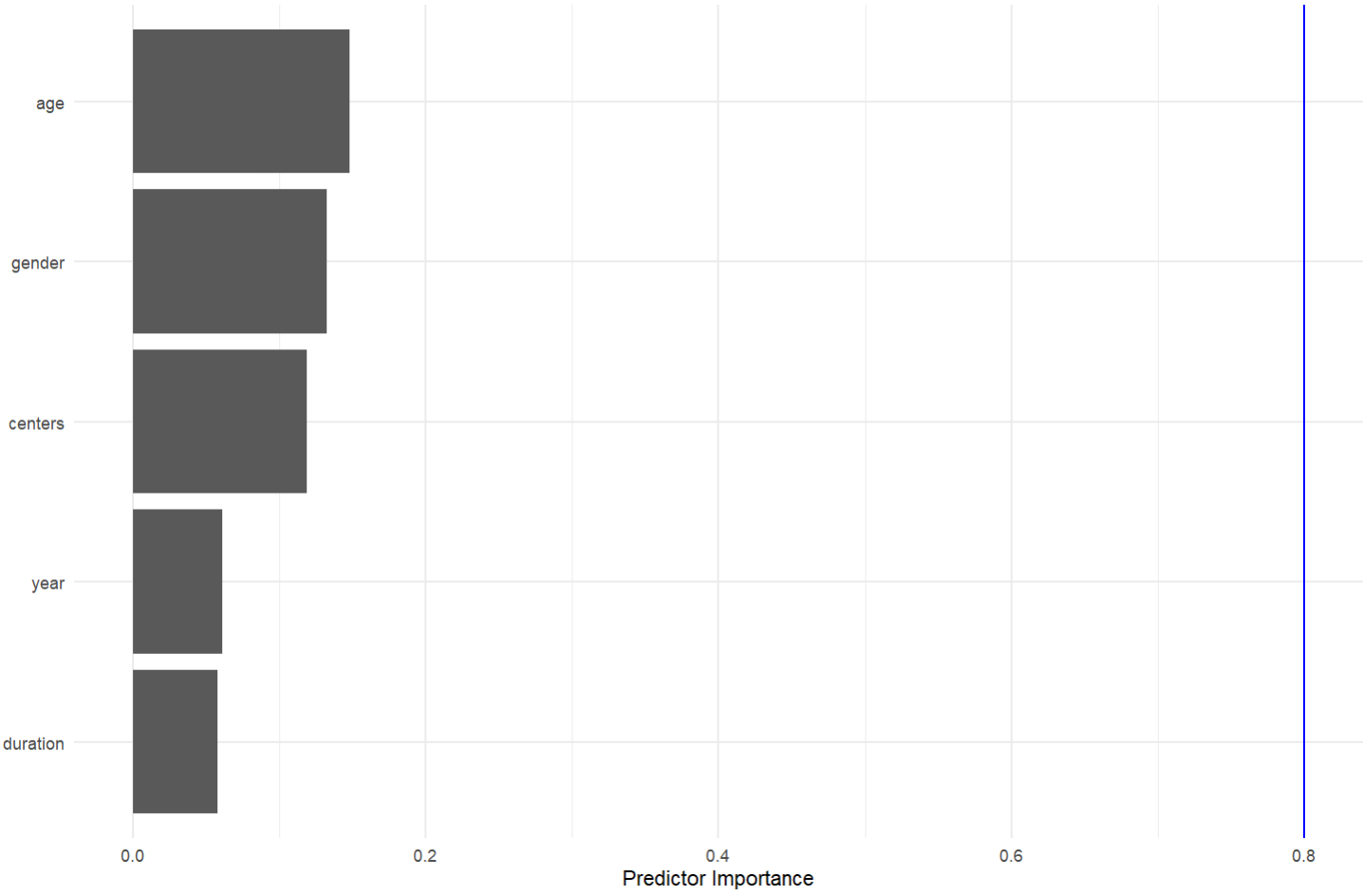


The possible combinations of best-fitting predictors, and the most important predictors, were tested by multi-model inference.
